# Down-Regulation of NDUFB9 Promotes Breast Cancer Cell Proliferation, Metastasis by Mediating Mitochondrial Metabolism

**DOI:** 10.1371/journal.pone.0144441

**Published:** 2015-12-07

**Authors:** Liang-Dong Li, He-Fen Sun, Xue-Xiao Liu, Shui-Ping Gao, Hong-Lin Jiang, Xin Hu, Wei Jin

**Affiliations:** 1 Department of Breast Surgery, Key Laboratory of Breast Cancer in Shanghai, Fudan University Shanghai Cancer Center, Shanghai, 200030, China; 2 Department of Oncology, Shanghai Medical College, Fudan University, Shanghai, 200030, China; 3 Department of Radiotherapy, Lishui Central Hospital, Zhejiang, 323000, China; The First Affiliated Hospital with Nanjing Medical University, CHINA

## Abstract

Despite advances in basic and clinical research, metastasis remains the leading cause of death in breast cancer patients. Genetic abnormalities in mitochondria, including mutations affecting complex I and oxidative phosphorylation, are found in breast cancers and might facilitate metastasis. Genes encoding complex I components have significant breast cancer prognostic value. In this study, we used quantitative proteomic analyses to compare a highly metastatic cancer cell line and a parental breast cancer cell line; and observed that NDUFB9, an accessory subunit of the mitochondrial membrane respiratory chain NADH dehydrogenase (complex I), was down-regulated in highly metastatic breast cancer cells. Furthermore, we demonstrated that loss of NDUFB9 promotes MDA-MB-231 cells proliferation, migration, and invasion because of elevated levels of mtROS, disturbance of the NAD^+^/NADH balance, and depletion of mtDNA. We also showed that, the Akt/mTOR/p70S6K signaling pathway and EMT might be involved in this mechanism. Thus, our findings contribute novel data to support the hypothesis that misregulation of mitochondrial complex I NADH dehydrogenase activity can profoundly enhance the aggressiveness of human breast cancer cells, suggesting that complex I deficiency is a potential and important biomarker for further basic research or clinical application.

## Introduction

Despite great achievements in clinical therapy, metastasis is still the leading cause of death in breast cancer patients. [1] A more comprehensive understanding of the cellular and molecular mechanisms that drive metastasis is vital for the development of more effective therapies. To initiate metastatic cells dissemination, it is necessary for cancer cells to acquire specific traits, including migration, invasion, and survival in the blood stream. [2]

Increasing evidence has supported the hypothesis that metastasis is under mitochondrial control. Valerie S. LeBleu demonstrated that circulating cancer cells (CTCs) exhibit enhanced mitochondria biogenesis and respiration.[3] Other studies have shown that CTCs can also be protected by aerobic glycolysis.[4] Additionally, playing a dual role in tumors, reactive oxygen species (ROS) are produced mainly by mitochondria, [5] and contributes to malignancy by participating in molecular and cellular events involving cytoskeletal rearrangements, regulation of signaling pathways and transcriptional activities that favor cell migration, invasion and anti-apoptosis.[6] In addition to mitochondrial DNA (mtDNA) mutations resulting from ROS that can lead to the progression of cancer, [7] altered mitochondrial DNA content is correlated with malignant potential in various cancers.[8]

In our previous study, we showed that MDA-MB-231HM cells demonstrated increased invasiveness compared to the parental MDA-MB-231 cell lines.[9] Due to the analogous genetic background of these cells, they provide an excellent model for screening potential metastasis-associated biomarkers and candidate therapeutic targets for triple negative breast cancer (TNBC). In this study, to reveal a panoramic view of the metastasis-related proteins in progressive TNBC, iTRAQ labeling followed by nanoscale high-performance liquid chromatography-tandem mass spectrometry (nano-HPLC-MS/MS) was applied to compare the whole-cell proteome profile of the two cell lines.[10] Our analysis identified NDUFB9 as a differentially expressed protein that was associated with the metastatic potential of TNBC.

NDUFB9 (NADH dehydrogenase (ubiquinone) 1 beta sub-complex, 9, 22kDa) is an accessory subunit of the mitochondrial membrane respiratory chain NADH dehydrogenase (complex I), [11] and is encoded by a nuclear gene.[12] Mutation of NDUFB9 can lead to complex I deficiency, [13] which has been reported to promote tumor metastasis.[14, 15] Additionally, SNP (rs7830235) associated with prostate cancer risk is located in the NDUFB9 gene.[16]

Using online “Kaplan Meier plotter” (KM plotter) database, in which updated gene expression data and survival information are supported for a total of 4142 breast cancer patients, we found that the majority of other subunits (NDUFB1-8/11) of the NADH dehydrogenase family had significant prognostic value (DMFS) for breast cancer patients [17] (S1 Fig **and Table C in** S1 File). However, NDUFB9 was not in the database. In the present study, we showed, for the first time, that NDUFB9 was a suppressor of MDA-MB-231 breast cancer cell proliferation, migration and invasion.

## Materials and Methods

### iTRAQ-nano-HPLC-MS/MS analyses

The cell lysates from parental MDA-MB-231 breast cancer cells and highly metastatic MDA-MB-231 cells (MDA-MB-231HM) were quantified using a Bradford assay, labeled with iTRAQ labeling reagents (Applied Biosystems), and digested with trypsin. The peptides were fractionated on a Waters ultra-performance liquid chromatography (UPLC) device, and the fractions were then separated by nanoscale high-performance liquid chromatography (nano-HPLC) (Eksigent Technologies) on a secondary reverse-phase (RP) analytical column. A Triple TOF 4600 mass spectrometer (MS) was operated in information-dependent data acquisition mode to switch automatically between MS and tandem MS (MS/MS) acquisition. The MS Data Converter from AB Sciex was used to extract the MS/MS spectra and to deconvolute the charge state.

### Cell culture

All of the breast cancer cell lines, normal breast MCF10A cells and HEK 293T cells were obtained from the American Type Culture Collection (Manassas, VA, USA) and maintained under conditions specified by the provider. All of the cells were cultured in a 5% CO_2_ incubator at 37°C.

### Western blot analysis

Whole-cell lysates were generated using the Pierce Tissue Protein Extraction Reagent (T-PER; Thermo Fisher Scientific Inc.) containing protease inhibitor cocktail tablets (Roche) and phosphatase inhibitors (Roche). In total, 30 μg of cell lysates was resolved by sodium dodecyl sulfate polyacrylamide gel electrophoresis (SDS-PAGE) and transferred to polyvinylidene fluoride (PVDF) membranes (Pall). The membranes were blocked in 5% milk or 5% bovine serum albumin and then incubated with various primary antibodies followed by the appropriate horseradish peroxidase (HRP)-conjugated secondary antibodies. Immunoreactive bands were identified using enhanced chemiluminescence, according to the manufacturer’s instructions, and the bands were quantified by densitometry. Antibodies used in this study are listed **in Table A and Table B in** S1 File.

### Quantitative real-time PCR

Total RNA was extracted with TRIzol reagent (Invitrogen Corporation) and reverse transcribed using the PrimeScript RT Reagent Kit (Perfect Real Time; TaKaRa Biotechnology). Subsequently, real-time PCR was performed with SYBR Premix Ex Taq (TaKaRa Biotechnology) using an ABI Prism 7900 instrument (Applied Biosystems). The following primer sequences were used in this study:

GAPDH, F 5’-GGTGGTCTCCTCTGACTTCAACA-3’

GAPDH, R 5’-GTTGCTGTAGCCAAATTCGTTGT-3’;

NDUFB9, F 5’-GGTGCGTCCAGAGAGACAAA-3’

NDUFB9, R 5’-ATCACCTTCCTTTCGGGCAG-3’;

### Plasmids and short hairpin RNA (shRNA)

Human NDUFB9 shRNAs and the negative control were purchased from GeneChem and expressed in the GV248 backbone. The target sequences were as follows:

shRNA-1: 5’-TCATCGTGGTGTATGACGT-3’;

shRNA-2: 5’-CCATCACTTCCAGCTACTA-3’.

### Lentivirus packaging and infection

Briefly, 293T cells were co-transfected with lentiviral vectors and the PCDH (or GV248), psPAX2 and pMD2G packaging vectors. Forty-eight hours after transfection, the viral supernatants were collected, filtered and concentrated by ultracentrifugation. Polybrene (Sigma-Aldrich, Natick, MA, USA) was added at a working concentration of 8 μg/ml. The cells were incubated with virus for 12 h, and then media containing fetal bovine serum (FBS) was added. After 48h, the infected cells were subjected to selection with 2 μg/ml puromycin for one week.

### Cell proliferation assays

Cell proliferation assays were performed using Cell Counting Kit-8 (Sigma-Aldrich). The cells were seeded in 96-well plates in media containing 10% FBS at 2,000 cells/well. At the indicated time points, each well was treated with the CCK-8 solution and the cells were incubated for 3 h at 37°C. The cell numbers were measured in a 96-well format plate reader (Tecan Sunrise, Switzerland) in triplicate by measuring the absorbance at a wavelength of 450 nm (OD450) every 24 h for 7 days.

### Cell cycle analysis via flow cytometry

For cell cycle analysis, 5×10^5^ cells were plated in a 6-well culture plate overnight. The cells were then incubated with serum-free medium for 24 h to synchronize cells at the G1/S boundary. Then cells were treated with fresh media containing 10% FBS for different times. Next, the cells were trypsinized, washed twice with cold PBS and fixed with cold 70% ethanol at -20°C overnight. The cells were then washed twice with PBS and incubated with 10 mg/ml RNase A, 400 mg/ml propidium iodide and 0.1% Triton X in PBS at room temperature (RT) for 30 m. Cells were subsequently analyzed by flow cytometry(BD Biosciences, San Jose, CA, USA).

### Transwell assays

Cells (5×10^5^ for the migration assay and 1×10^6^ for the invasion assay) were plated in the top chamber of a non-coated membrane or Matrigel-coated transwell chambers (BD Biosciences) in media without FBS. Media supplemented with serum (twofold versus normal for MDA-MB-231 and fivefold versus normal for MCF-10A) was used as a chemoattractant in the lower chamber. The cells were incubated for 12-36h (12h for MDA-MB-231 migration, 20h for MDA-MB-231 invasion and 36h for MCF-10A migration), and the cells that did not migrate through the pores were removed with a cotton swab. The cells on the lower surface of the membrane were stained with methanol and 0.1% crystal violet and then counted.

### Kinetic Wound-Healing Assay

Breast cancer cells (3.5×10^4^) were plated on 96-well plates (Essen Image Lock, Essen Instruments), and a wound was scratched with wound scratcher (Essen Instruments). Wound confluence was monitored with Live-Cell Imaging System and software (Essen Instruments). Wound closure was observed every 2 hours for 28 hours by comparing the mean relative wound density of at least three biological replicates in each experiment.

### Mitochondrial ROS Determination

Cells were harvested, washed and resuspended in 5 μM MitoSOX (Invitrogen Corp.) working solution for 15 min at 37°C. After centrifuging, cells were resuspended in 500 μL PBS and analyzed for mitochondria ROS production by flow cytometry (BD Biosciences, San Jose, CA, USA).

### NAD^+^/NADH analysis in cell

Intracellular NAD^+^ and NADH were analyzed independently in extracts of whole cells (3.0×10^4^) seeded in 96-well black, clear bottom plate (Corning, USA) the day before. Concentrations were determined using a NAD^+^/NADH fluorescence detection kit (Abcam) according to manufacturer’s instructions.

### Mt-DNA

Mt-DNA content was determined by quantitative real-time PCR of mtDNA extracted from 2 × 10^7^ cells using Mitochondrial DNA Isolation Kit (BioVision Milpitas, CA USA). Real-time PCR was performed with SYBR Premix Ex Taq (TaKaRa Biotechnology) using an ABI Prism 7900 instrument (Applied Biosystems). The primer sequences used in this study are as follows:

Primer-1 F: 5’-ATACCAAACGCCCCTCTTCG-3’

Primer -1 R: 5’-TGTTGAGGTTGCGGTCTGTT-3’

Primer -2 F: 5’-GGAACTACTCCCACCCTGGA-3’

Primer -2 R: 5’-GGGTTATGGCAGGGGGTTTT-3’

Primer -3 F: 5’-AACGTTATCGTCACAGCCCA-3’

Primer -3 R: 5’-TTGTTTATGCGGGGAAACGC-3’

### Glucose uptake assay

The cells were seeded at a density of 2 × 10^4^ cells/well in 96-well black, clear bottom plate (Corning, USA) and disposed by Glucose Uptake Cell-Based Assay Kit (Cayman, Ann Arbor, Michigan, USA) according to the manufacturer's operations. Cells were measured using a plate reader (excitation/emission = 485 nm/535 nm).

### Lactate production assay

A total of 3 × 10^5^ cells were cultured in a 6-well plate for 72 h. Supernatant was collected and was deproteinized due to high LDH content. Supernatant was collected and measured by lactate assay kit from BioVision (Mountain View, CA, USA).

### Statistical analysis

The results were reported as the mean ± SD or mean ± SEM, as indicated in the figure legends. The results were analyzed using PRISM 5.0 (GraphPad Software Inc., San Diego, CA, USA). P values < 0.05 were considered statistically significant.

## Results

### Highly metastatic breast cancer cells have low levels of NDUFB9 expression

**Fig 1:**
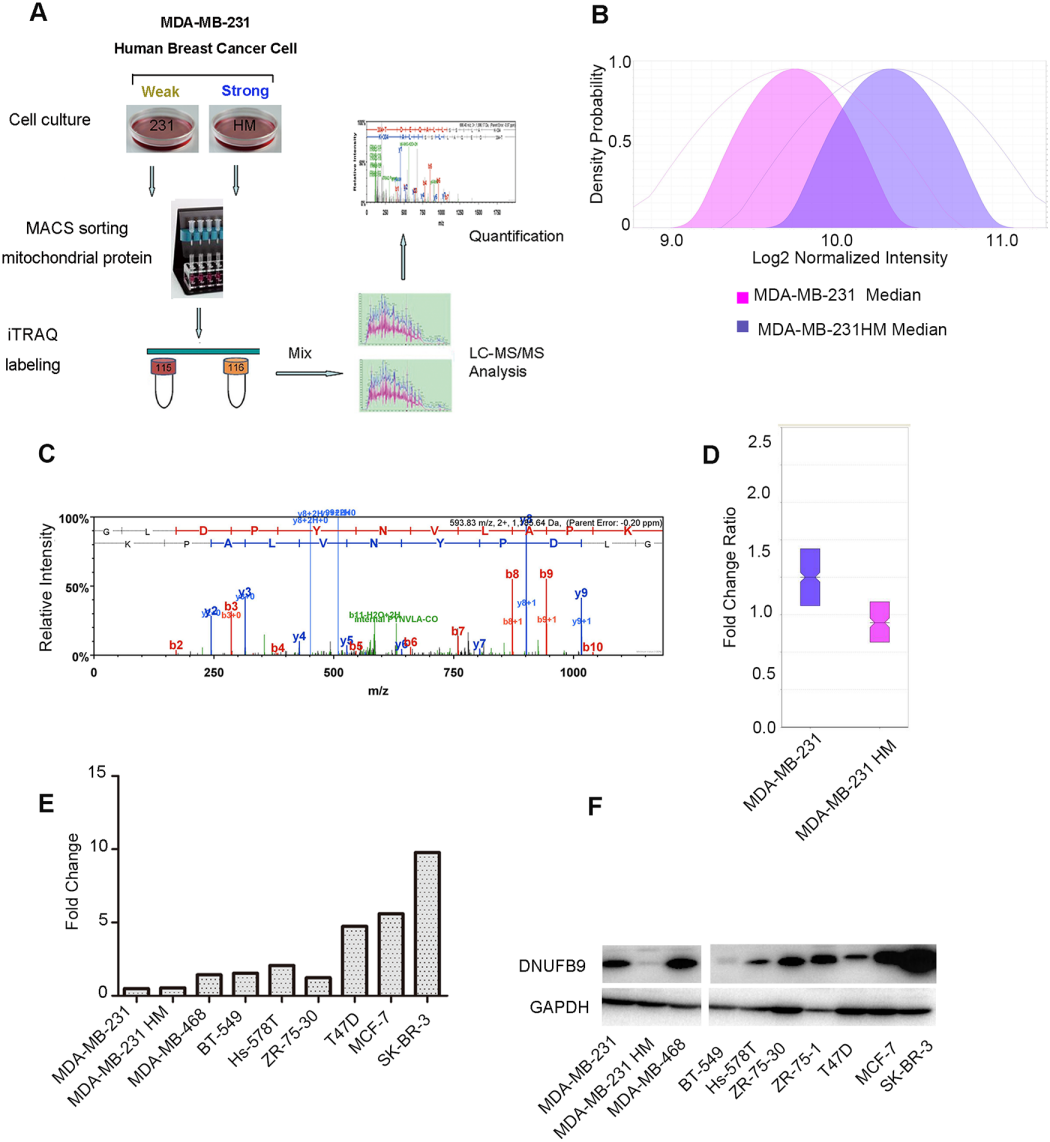
Low expression levels of NDUFB9 in highly metastatic breast cancer cells. (**A**) Schematic overview of iTRAQ. (**B**) The normalized median density of MDA-MB-231 and MDA-MB-231HM. (**C**) The spectrum of NDUFB9 obtained from MS. (**D**) The fold change ratio in the MDA-MB-231 and MDA-MB-231HM breast cancer cell lines by iTRAQ labeling based on quantitative proteomic analyses. (**E,F**) Protein and mRNA expression of NDUFB9 in breast cancer cell lines. Results are representative of 3 independent experiments.

Using the iTRAQ labeling method in our model system, we found that NDUFB9 expression was slightly suppressed in MDA-MB-231HM cells compared to MDA-MB-231 cells (Fig 1A–1D). In addition to these two cell lines, we also detected the general gene expression trend in a panel of other breast cancer cell lines. In accordance with the results observed in our iTRAQ experiments, on the whole, the weakly metastatic cell lines (MCF-7, T47D, ZR-75-30, and SK-BR-3) expressed NDUFB9 at higher levels, but highly metastatic cell lines (BT-549, Hs578T, MDA-MB-468, MDA-MB-231, and MDA-MB-231HM) expressed it at lower levels (Fig 1E and 1F), even if with the exception of a few individual cases. Taken together, these data suggest that NDUFB9 might repress tumor metastasis.

### Knockdown of NDUFB9 promotes MDA-MB-231 proliferation

**Fig 2:**
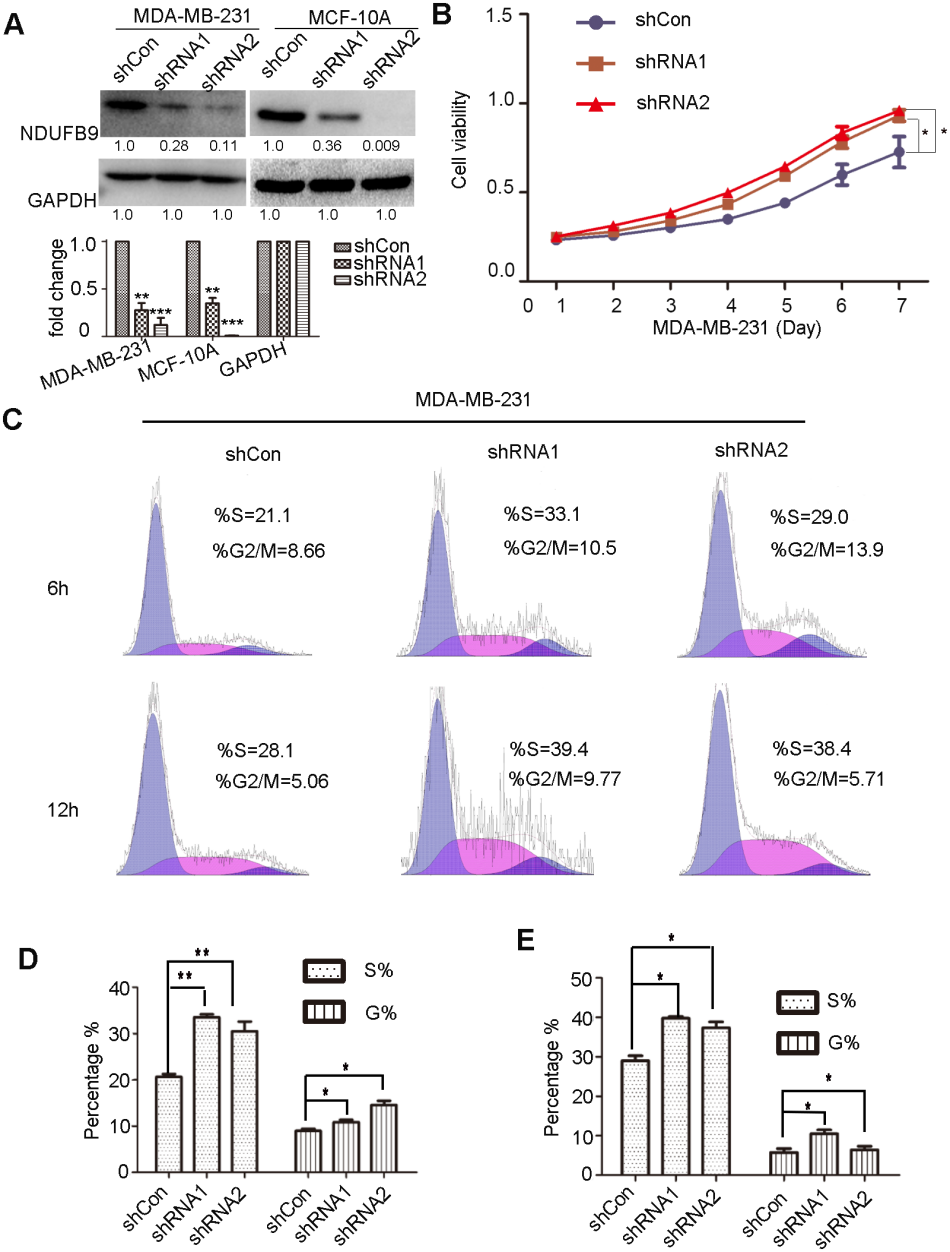
Knockdown of NDUFB9 promoted MDA-MB-231 proliferation. **(A)** Knockdown efficiency of NDUFB9 in MDA-MB-231 and MCF-10A cells analyzed by Western blot and the below panel shows histograms of the results. **(B)** CCK-8 proliferation assays in MDA-MB-231 shCon and shNDUFB9 cells. **(C)** Cell cycle analysis in MDA-MB-231 shCon and shNDUFB9 cells at 6h and 12h after cells were synchronized at the G1/S boundary. **(D, E)** Histograms of the S and G2/M phases of the cell cycle for 6 h and 12 h respectively. Results above are repeated at least 3 times. Data were presented as the mean ± SD (Student’s t-test, n≥3; *P < 0.05, **P < 0.01 and ***P < 0.001).

To further investigate if NDUFB9 functions in cancer cell metastasis, we employed two independent shRNAs targeting NDUFB9 in MDA-MB-231 cell lines, and an empty vector (shCon) served as a control. Western blot results demonstrated that NDUFB9 expression was strongly down-regulated by both shRNA constructs (Fig 2A). A significant increase in the proliferation rate was observed after NDUFB9 knockdown in MDA-MB-231 cells compared to shCon MDA-MB-231 cells according to the CCK-8 assay (Fig 2B). Then flow cytometry was used to examine the cell cycle changes. In accordance with the proliferation results, suppression of NDUFB9 expression resulted in a higher percentage of cells in S phase and G2 phase at 6 and 12 hours after cell cycle synchronization respectively (Fig 2C–2E). These results suggest a potential role for the loss of NDUFB9 in MDA-MB-231 cells in promoting cell proliferation.

### Loss of NDUFB9 expression results in enhancement of MDA-MB-231 cells migration and invasion and MCF-10A cells migration

**Fig 3:**
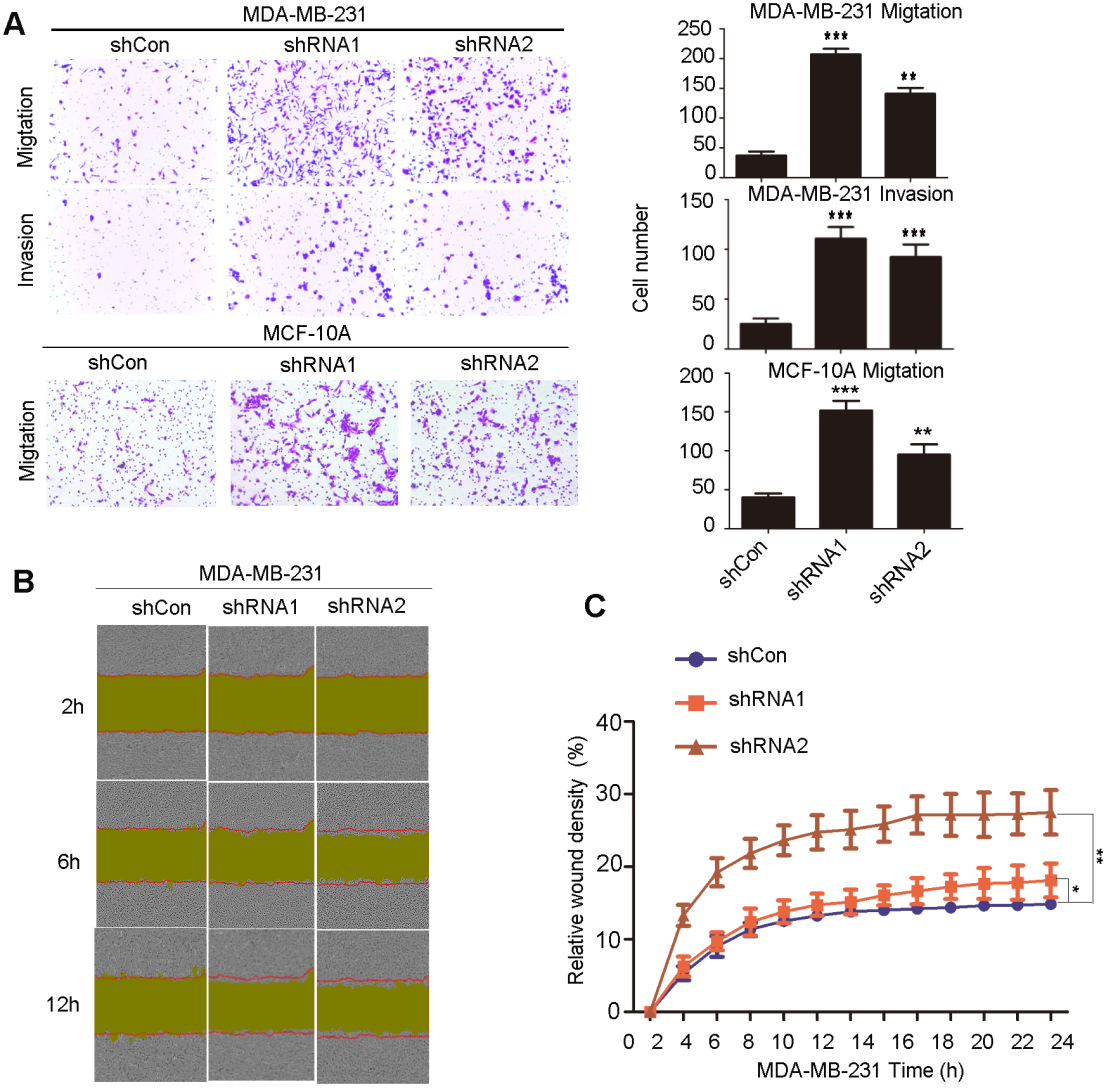
Loss of NDUFB9 expression resulted in enhancement of MDA-MB-231 cell migration and invasion. **(A)** The migration and invasion ability of MDA-MB-231 and migration of MCF-10A were evaluated with a transwell assay. The left panels show images of representative fields (100× magnification) of invasive cells, and the right panel shows histograms of the results. **(B)** Effects of NDUFB9 on the migration of MDA-MB-231 cells in a wound-healing assay. The red lines indicate the initial scratch wound location, and the yellow area shows the scratch wound mask. Images were captured at the indicated times after wounding. **(C)** The effect of NDUFB9 on the percent of relative wound density at the indicated time point. Results above are repeated at least 3 times. Data were presented as the mean ± SD (Student’s t-test, n≥3; **P < 0.01, and ***P < 0.001).

To further investigate if loss of NDUFB9 affects cell migration and invasion, we performed transwell migration and transwell Matrigel invasion assays. As expected, in MDA-MB-231 cell line, both groups with suppressed NDUFB9 expression showed a significantly enhanced ability to migrate through the basement membrane and invade the Matrigel membrane compared to the control group (Fig 3A). The migration ability of MCF-10A cells was also elevated after downregulation of NDUFB9 (Fig 3A). Moreover, we used a wound-healing assay to demonstrate that the NDUFB9 knockdown group had higher migration rates, compared to the control group (Fig 3B and 3C). Thus, these data demonstrate that loss of NDUFB9 promotes the migration and invasion of MDA-MB-231 and MCF-10A cells.

### Depletion of NDUFB9 disturbs mitochondrial metabolism

**Fig 4:**
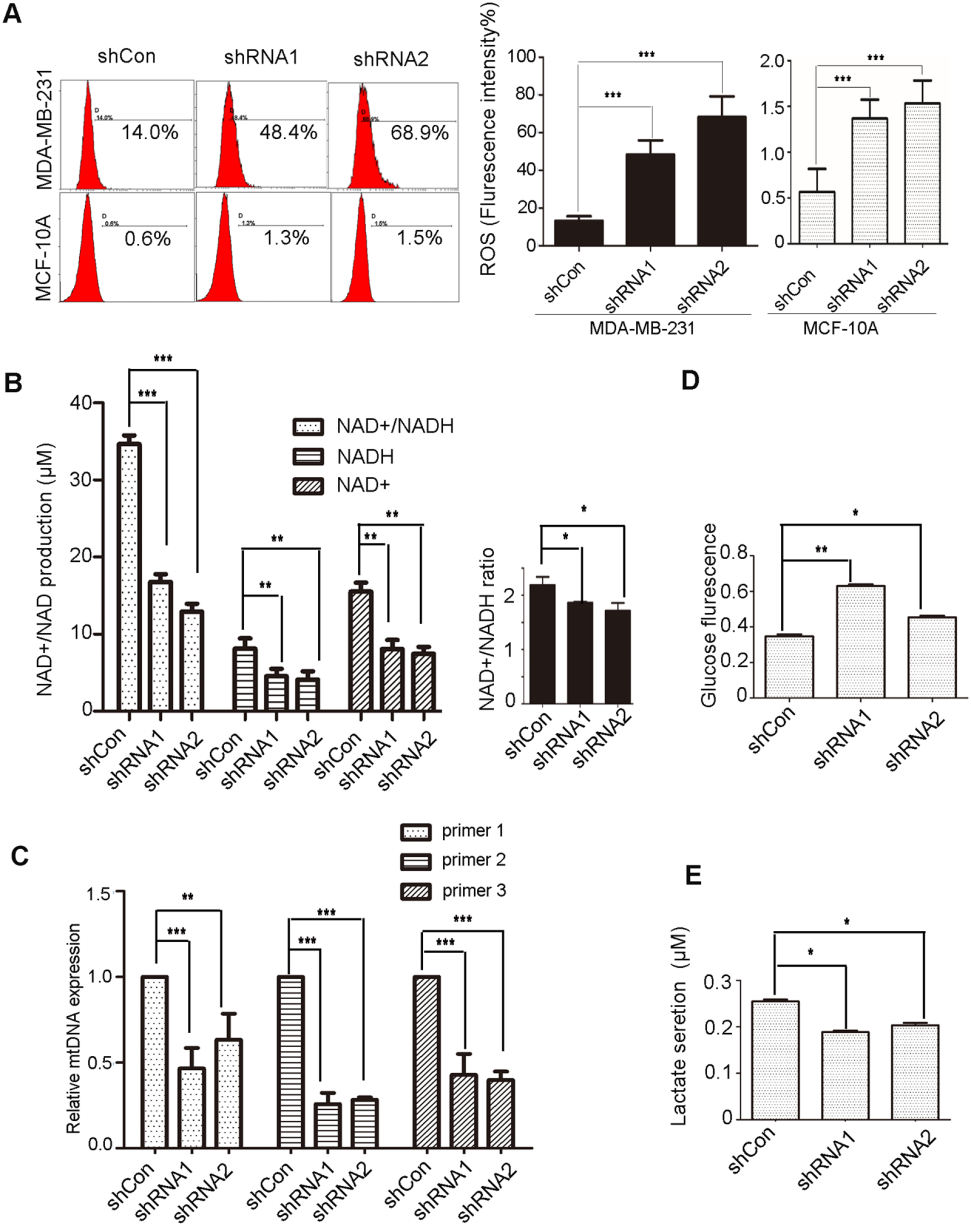
Depletion of NDUFB9 altered mitochondrial metabolism. **(A)** FACS analysis (left) and statistical data (right) of mitochondrial ROS generation in MDA-MB-231 and MCF-10A cells. **(B)** Intracellular NAD^+^, NADH, total NAD^+^/NADH and the ratio of NAD^+^/NADH were analyzed independently in extracts from whole cells in the NDUFB9 knockdown and control groups. **(C)** mt-DNA content was determined by quantitative real-time PCR of mtDNA extracted from MDA-MB-231 cells, using three pairs of mtDNA primers (primer-1, primer-2, and primer-3). **(D, E)** Glucose uptake and lactate secretion were assessed in NDUFB9 knockdown or control MDA-MB-231 cells. Results above are repeated at least 3 times. Data were presented as the mean ± SD (Student’s t-test, n≥3; **P < 0.01, and ***P < 0.001).

Previous studies have reported that mtDNA mutations, which partially inactivated ETC complex I and subsequently led to the elevation of mitochondrial reactive oxygen species (mtROS) and nonlethal reduction of NAD^+^ levels promoted tumor metastasis.[15, 18] Given that mutation of NDUFB9 can lead to complex I deficiency, [13] we investigated mtROS production, NAD^+^/NADH levels, and mtDNA content in NDUFB9 knockdown and control cells. As expected, decreased NDUFB9 expression resulted in significantly higher levels of mtROS and lower levels of NAD^+^/NADH (total), NAD^+^, and NADH, but suppression of NDUFB9 resulted in only a slightly lower ratio of NAD^+^/NADH. Additionally, in MCF-10A cells, downregulation of NDUFB9 also led to overproduction of mtROS. (Fig 4A and 4B).

Several studies had demonstrated that depletion of mtDNA induces prostate colorectal and osteosarcoma cancer progression.[19–21] Similarly, we found that mtDNA content was remarkably reduced after NDUFB9 knockdown in MDA-MB-231 cells; and was accompanied by more traits that are associated with tumor progression, compared to the control cells (Fig 4C).

Because the processes of aerobic respiration and anaerobic respiration occur in the mitochondria, complex I functions as the recipient of aerobic and anaerobic glycolysis metabolite. Thus we evaluated glucose uptake and lactate secretion to assess the effects of complex I on the metabolic pathway. The results showed that glucose uptake was slightly elevated, whereas lactate secretion was decreased, in response to NDUFB9 suppression compared to the control (Fig 4D and 4E).

Taken together, these results suggested that a loss of NDUFB9 lead to mitochondrial metabolism disorders.

### Suppression of NDUFB9 expression induces EMT and activates the AKT/mTOR/p70S6K signaling pathway

**Fig 5:**
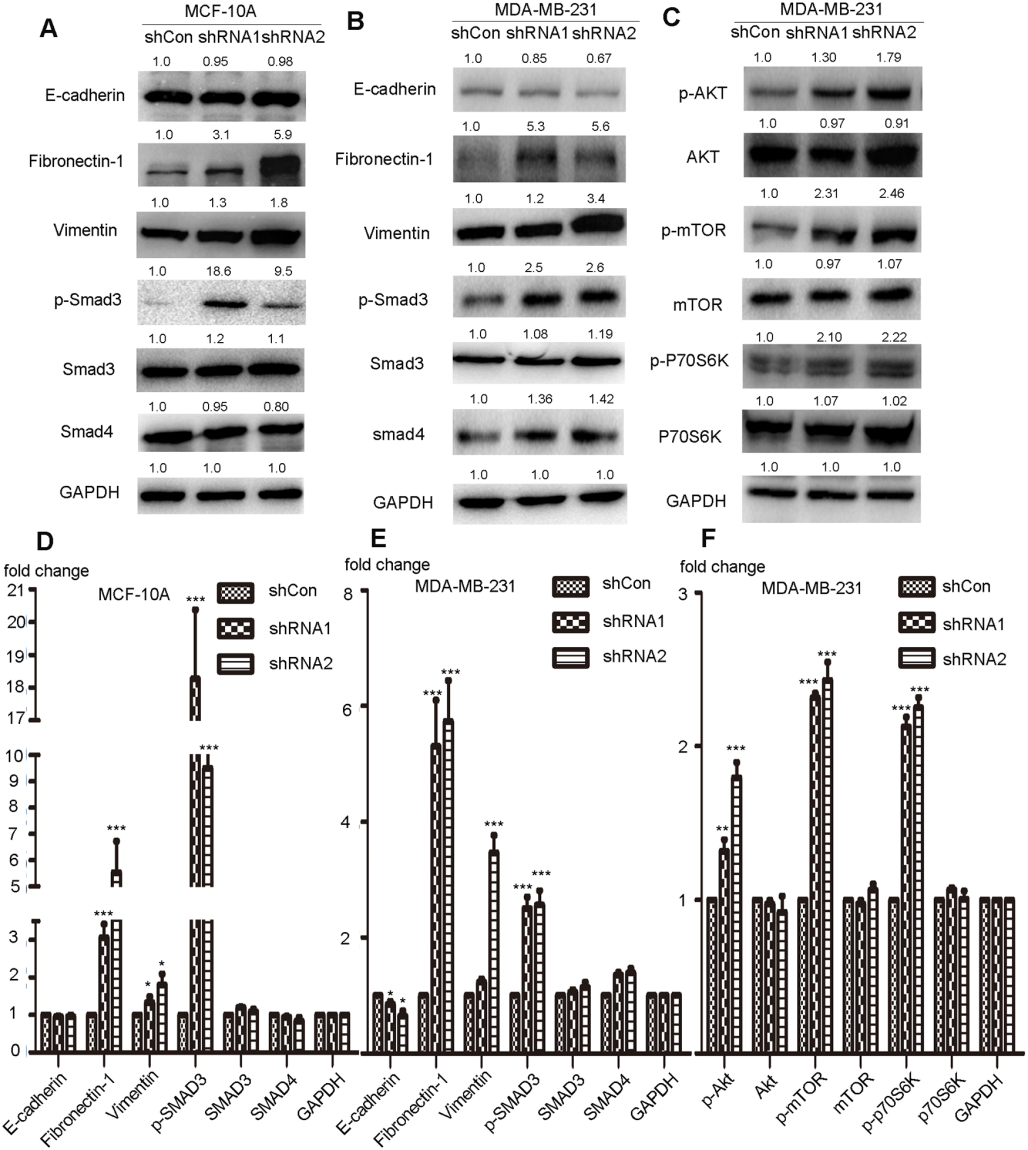
Suppression of NDUFB9 expression induced EMT and activated the AKT/mTOR/p70S6K signaling pathway. (A-B) Immunoblotting analysis of E-cadherin, Fibronectin-1 (FN-1), Vimentin, phosphorylated SMAD3, SMAD3 and SMAD4 in MCF-10A and MDA-MB-231 cells transfected with NDUFB9-specific or control shRNA. GAPDH was used to normalize for equal loading. (C) Expression levels of phosphorylated Akt (S129), total Akt, phosphorylated mTOR(S2448), total mTOR, phosphorylated p70S6K and p70S6K were examined by immunoblotting in MDA-MB-231. Results above are representative of 3 independent experiments. (D-F) Histograms of the results from A, B and C respectively. Data were presented as the mean ± SD (Student’s t-test, n≥3; **P < 0.01, and ***P < 0.001).

EMT, which plays essential roles in development and wound healing, is also considered as a key step in cancer metastasis.[22] Thus, we sought to determine if loss of NDUFB9 induces EMT in MDA-MB-231 cells by examining the expression level of several epithelial and mesenchymal markers. As shown in Fig 5B and 5E, NDUFB9 knockdown cells exhibited decreased expression of the epithelial marker: E-cadherin; and increased expression of the mesenchymal markers: Vimentin and Fibronectin-1 (FN-1); compared to the control. Additionally, we also detected that, in MCF-10A, Vimentin and Fibronectin-1 (FN-1) upregulated in response to downregulation of NDUFB9 (Fig 5A and 5D).

mTOR activity can also be affected by complex I; [15] and the Akt/mTOR signaling pathway is an upstream regulator of proliferation, EMT and cancer metastasis.[23–27] Therefore, we investigated if the Akt/mTOR signaling pathway is activated by loss of NDUFB9 in MDA-MB-231 cells. Down-regulation of NDUFB9 markedly enhanced the phosphorylation of Akt (S129), mTOR (S2448) and p70S6K in MDA-MB-231 cells (Fig 5C and 5F). As an up-stream regulator of EMT, Smad3 presented more phosphorylation significantly after loss of NDUFB9 both in MDA-MB-231 and MCF-10A (Fig 5A, 5B, 5D and 5E).

## Discussion

Defective oxidative phosphorylation has a crucial role in the attenuation of mitochondrial function, which contributes to tumourgenesis and cancer progression.[28] Moreover, mitochondria contain a variety of proteins that are necessary for both the promotion and prevention of cell death. Thus, mitochondria play a pivotal role in deciding cell fate and are becoming potentially a promising area of research for cancer diagnosis and therapy. However, the clinical applications for the role of dysfunctional mitochondria in cancer progression are limited.[29]

As one of the 45 subunits constituting complex I, loss of NDUFB9 results in complex I deficiency and disturbs the normal process of electron transfer, thereby contributing to an unbalance of NAD^+^/NADH, a reduction of NAD^+^ and a reduction of NADH. Together with other mechanisms, these changes ultimately promote MDA-MB-231 cells migration and invasion. In addition, we also observed that the majority of other subunits (NDUFB1-8/11) from the NADH dehydrogenase family have significant prognostic value (DMFS) in breast cancer patients (S1 Fig), indicating the considerable clinical value.

Complex I, which is embedded in the inner mitochondrial membrane (IMM) and consists of 45 subunits, oxidizes NADH, reduces ubiquinone, and shuttles electrons to coenzyme Q.(30) Through the electron transport chain (ETC) in complex I, redox activity regenerates the NAD^+^ pool in the mitochondrial matrix.[30] Several studies have demonstrated that ETC overload or partial ETC inhibition, promote superoxide-dependent tumor cell migration, invasion, clonogenicity, and metastasis, [31] as well as a reduction of NAD^+^ levels and disturbance of the NAD^+^/NADH balance, rendering tumor cells more aggressive. However, enhancement of the NAD^+^/NADH balance through treatment with NAD^+^ precursors inhibits tumorigenicity and metastasis.[15, 32, 33]

It is widely reported that increased production of mitochondrial reactive oxygen species (mtROS) can promote tumor metastasis through multiple mechanisms, including inducing angiogenesis, EMT, cell migration signaling pathways, cell invasion signaling pathways, mtDNA mutation or mtDNA damage.[6, 34–36] Several polymorphic sequence variations and a plethora of somatic mutations have been identified in mitochondrial DNA (mtDNA), [7, 18] Moreover, the identification of altered mtDNA copy number has been reported in a broad range of primary human cancers, underlining that changes to mtDNA content be a pivotal factor inducing persistent mitochondrial deficits and eventually contributing to cancer pathogenesis and progression.[37, 38] Although the increase of mtDNA content have been observed in a wide range of cancers, decreased mtDNA levels have been detected in breast tumor tissues and peripheral blood consistently in a wide variety of studies.[39, 40] However, the causes that induce mtDNA changes and the functional significance of different mtDNA amounts in the tumorigenic process remain largely unknown.[41]

It has been reported that signaling through the AKT/mTOR/p70S6K pathway, which plays a vital role in regulating apoptosis and autophagy in cells, [38] controls migration and invasion of cells. Moreover, anti-cancer drugs inhibiting the AKT/mTOR/p70S6K axis putatively reduce cell migration and invasion.[39] Increasing evidence has suggested that EMT is a crucial step in tumor metastasis because it enables tumor cells to migrate and invade the surrounding stroma and spread to distant organs.[40] A role for elevated mTORC1 and mTORC2 activity in regulating the EMT, motility, and metastasis of colorectal cancer cells via RhoA and Rac1 signaling has also been demonstrated.[41] Additionally, there is evidence that supports the ROS-dependent activation of the Akt/mTOR pathway as a strategy adopted by cancer cells to reduce onconase (ONC)-mediated cytotoxic autophagy stimulation, [42] and EMT in mammary epithelial cells can be induced by ROS through the NF-kB-dependent activation of Snail.[43]

Our data provides new clues on the relationship among complex I deficiency, the Akt/mTOR pathway, EMT and cancer metastasis. Based on this study we can speculate that loss of NDUFB9 induced complex I deficiency, which triggered an NAD^+^/NADH imbalance, and oxidative stress in MDA-MB-231 cells. These changes reduced mtDNA levels and activated the Akt/mTOR signaling pathway, which subsequently induced EMT and elevated cell migratory and invasive capabilities, thus conferring MDA-MB-231 cells with a metastatic phenotype. To consolidate such a relationship, more efforts, including in vivo assays and cell line enlarging, are still needed to further explore the loss of the NDUFB9-mediated regulatory network in breast cancer cells.

Although no well-documented signaling pathway links have been established to date, our findings dropped a hint that complex I deficiency, including subunits dysfunction, unbalanced NAD^+^/NADH, and reduction of mtDNA content might serve as a potential and important biomarker to guide further research or clinical treatments with specific targeted therapies aimed at ROS and the Akt/mTOR pathway.

## Supporting Information

S1 Fig Kaplan—Meier survival curves of other subunits of complex I. (TIF)

S1 File Antibodies used in this study and Complex I subunits Affymetrix IDs. (DOC)

## Abbreviations

NDUFB9: NADH dehydrogenase (ubiquinone) 1 beta sub-complex, 9, 22kDa
NADH: Nicotinamide Adenine Dinucleotide Hydrogen
TNBC: Tripe-negative Breast Cancer
DMFS: Distant Metastasis Free Survival
ROS: Reactive Oxygen Species
ETC: Electron Transport Chain
EMT: Epithelial-Mesenchymal Transition
