# Supplementary figures and images for: Down-Regulation of NDUFB9 Promotes Breast Cancer Cell Proliferation, Metastasis by Mediating Mitochondrial Metabolism
Source: PLoS One. 2015 Dec 7;10(12):e0144441. doi: 10.1371/journal.pone.0144441 (PMC4671602; doi:10.1371/journal.pone.0144441)

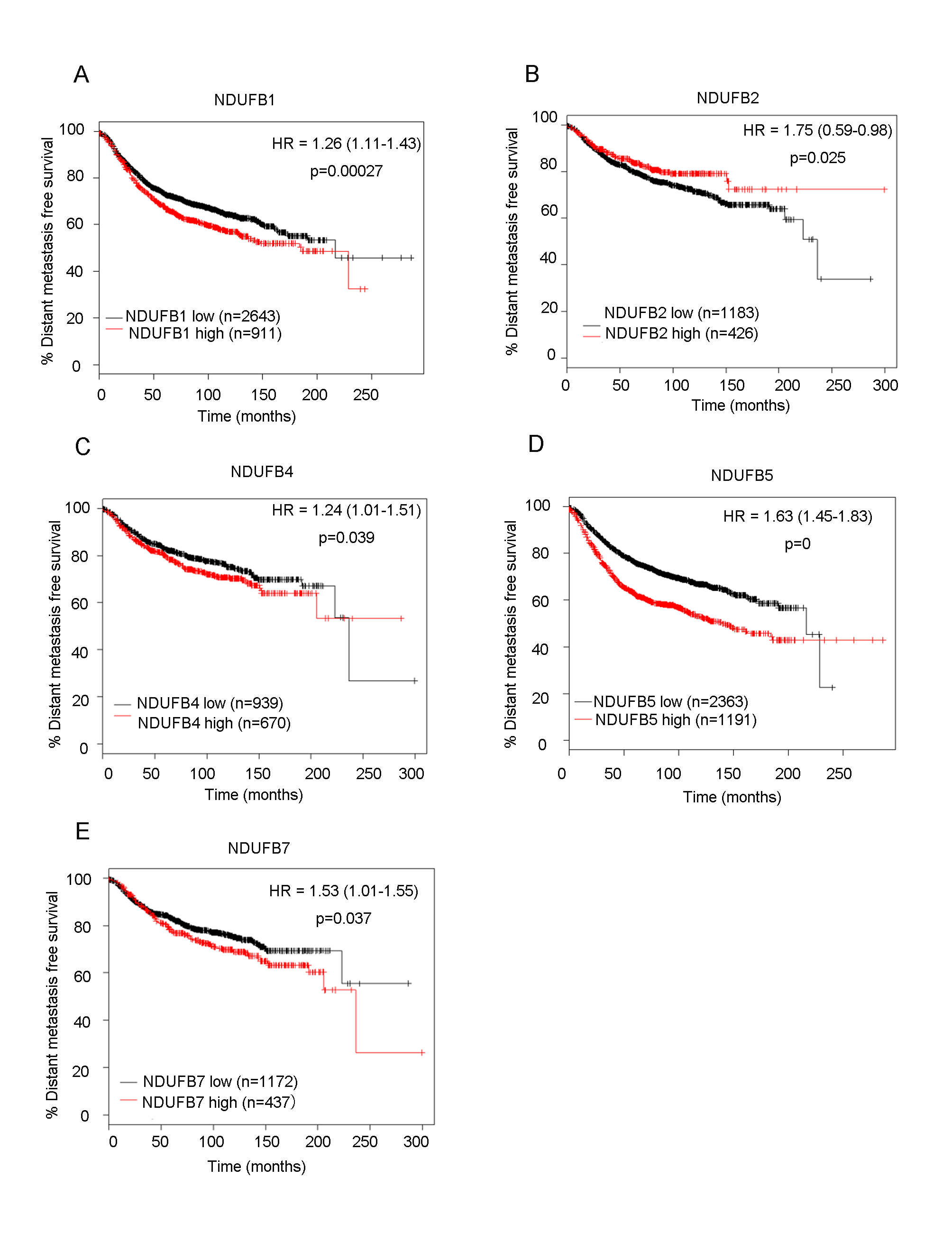

Supplement: S1 Fig — (TIF) [file pone.0144441.s001.tif]
