# Supplementary material for: Down-Regulation of NDUFB9 Promotes Breast Cancer Cell Proliferation, Metastasis by Mediating Mitochondrial Metabolism
Source: PLoS One. 2015 Dec 7;10(12):e0144441. doi: 10.1371/journal.pone.0144441 (PMC4671602; doi:10.1371/journal.pone.0144441)
Supplement: S1 File — (DOC) [file pone.0144441.s002.doc]

Table A: Primary antibodies used in this study

| Primary source | Antibody Clone | Dilution | Company |
| --- | --- | --- | --- |
| NDUFB9 | Rabbit polyoclonal | 1:100 | Santa Cruz |
| p-AKT | Rabbit monoclonal | 1:1000 | Epitomics |
| AKT | Rabbit monoclonal | 1:1000 | Epitomics |
| p-mTOR | Rabbit monoclonal | 1:2000 | Abcam |
| mTOR | Rabbit monoclonal | 1:2000 | Abcam |
| p70S6K | Rabbit polyoclonal | 1:1000 | Protein Tech |
| p-p70S6K | Rabbit polyoclonal | 1:1000 | Abcam |
| E-cadherin | Rabbit mAb | 1:1000 | CST |
| Vimentin | Rabbit monoclonal | 1:1000 | Protein Tech |
| Fibronectin | Mouse polyclonal | 1:1000 | Protein tech |
| p-Smad3(S423+S425) | Rabbit mAb | 1:2000 | Abcam |
| p-Smad4 | Rabbit mAb | 1:1000 | Protein tech |
| Cleaved PARP | Rabbit mAb | 1:1000 | Abcam |
| GAPDH | Rabbit mAb | 1:10000 | Protein tech |

Table B: Secondary antibodies used in this study

| Secondary Antibody | Dilution | Company |
| --- | --- | --- |
| Anti-mouse | 1:3000-1:5000 | Protein tech |
| Anti-rabbit | 1:3000-1:5000 | Protein tech |

Table C: Complex I subunits and their Affymetrix IDs

| Gene symbols | The Affymetrix IDs |
| --- | --- |
| NDUFB1 | 206791_s_at |
| NDUFB2 | 218200_s_at |
| NDUFB2 | 218201_at |
| NDUFB4 | 218226_s_at |
| NDUFB5 | 203621_at |
| NDUFB7 | 202839_s_at |
